# Tongue volume in spinal and bulbar muscular atrophy (SBMA): an AI-assisted automatic MRI analysis

**DOI:** 10.1007/s00415-026-13921-y

**Published:** 2026-06-07

**Authors:** Angela Rosenbohm, Ina Vernikouskaya, Anastasia Nosanova, Nam Nguyen-Younossi, Karl Georg Haeusler, Jochen Weishaupt, Volker Rasche, Hans-Peter Müller, Jan Kassubek

**Affiliations:** 1 Department of Neurology, University Hospital Ulm, Oberer Eselsberg 45, 89081 Ulm, Germany; 2 Department of Internal Medicine II, Ulm University Medical Center, Ulm, Germany; 3 German Center for Neurodegenerative Diseases (DZNE), Ulm, Germany; 4 Core Facility Small Animal MRI, University of Ulm, Ulm, Germany

**Keywords:** Magnetic resonance imaging, Tongue, Muscle volume, Motor neuron disease, Kennedy disease, Volumetry, Neuroimaging

## Abstract

Atrophy of the tongue muscle without severe dysarthria is one of the clinical hallmarks of spinal and bulbar muscular atrophy (SBMA), a motor neuron disease caused by an androgene receptor defect. An operator-independent AI-based automatic segmentation of the tongue was applied to 3-D MRI data of the head in SBMA in order to quantify the tongue atrophy. Thirty-nine patients with SBMA and 51 age-matched healthy controls underwent MRI which were used for tongue volume quantification. A single triplanar convolutional neural network of U-Net architecture trained on axial, coronal, and sagittal planes was used for the segmentation of the tongue in MRI scans of the head, the resulting volumes were processed slice-wise across the three orientations and corrected for age. At the group level, a significant atrophy of the tongue was observed in SBMA when compared to controls (p < 0.05). Atrophy correlated well with total SBMA-functional rating scale and even more with bulbar subscores. In summary, the study employed an AI-assisted advanced imaging analysis to quantify the tongue morphology in individuals with SBMA in correlation to clinical bulbar function, suggesting this approach as a potential biomarker for disease assessment.

## Introduction

Spinal and bulbar muscular atrophy (SBMA), also known as Kennedy Disease, is a combined motor neuron/muscle disease with adult onset, resulting from an abnormal expansion of an unstable CAG trinucleotide repeat in the androgen receptor (*AR*) gene [1, 2]. This condition profoundly affects neuromuscular function, leading to the degeneration of spinal motor neurons and significant muscle involvement [3]. The damage to motor cranial nerves in SBMA leads to bulbar involvement and subsequent atrophy of the tongue muscle [4]. Changes in tongue muscles are a frequent initial manifestation of the disease. Tongue morphology is generally assumed to be directly related to the resulting speech (dysarthria) and swallowing (dysphagia) difficulties, although in SBMA, the atrophy of the tongue muscle is also observed without severe dysarthria.

The measurement of tongue volume is a valuable advance for understanding disease progression and functional impairments in motor neuron diseases (MND) and has so far been evaluated in ALS [5, 6], but not in SBMA. The tongue’s role in articulation and deglutition makes its assessment vital for understanding the disease’s progression and its implications on patients’ quality of life. Studies have indicated that the degeneration of motor neurons directly affects the neuromuscular coordination necessary for swallowing and speaking, with dysarthria and dysphagia as common symptoms that significantly impair daily activities and reduce quality of life [7, 8].

The primary objective of this study was the assessment and quantification of tongue musculature atrophy in individuals with SBMA in order to assess the relationship between tongue mechanics and clinical outcomes.

## Methods

Inclusion criteria were genetically confirmed diagnosis of SBMA and written consent to participate in the study. Patients opting to participate had to be able to undergo MRI examination. We recruited male SBMA patients from the outpatient clinic of the Department of Neurology, University Hospital Ulm, Germany. Forty male patients with genetically confirmed SBMA entered the study; after excluding one MRI for image quality/tongue movements, n = 39 could be evaluated. Information about individual disease history and characteristics, comorbidities, neurological, and cardiovascular symptoms were obtained in structured personal interviews and clinical examinations.

Ethical approval of the Ethics Committee of Ulm University, Germany was obtained for the study (reference no. 258/22). Written informed consent was obtained from each participant.

The SBMA-FRS (SBMA functional rating scale) in total (maximum 56 points) and in the domains relating to bulbar dysfunction (maximum 20 points) were compared to tongue volumes, as well as height, weight and BMI, disease duration, age at first symptom, and CAG repeat length.

AI-assisted automatic MRI-based tongue volume evaluation was performed to determine the atrophy of the tongue. This approach was based on the methodology developed in a previous study [5]. The AI-assisted measurement utilizing a convolutional neural network (CNN) architecture, aimed to automate the segmentation of tongue volumes from MRI data. For this study, high-resolution T1-weighted Magnetization Prepared Rapid Gradient Echo (MPRAGE) data with 144 slices in sagittal orientation (256 × 256 pixels, slice thickness 1.2 mm (without slice gaps), pixel size 1.0 mm × 1.0 mm, echo time was 4.2 ms, repetition time was 1640 ms) were acquired on a 1.5 Tesla Magnetom Symphony (Siemens Medical, Erlangen, Germany). The CNN was trained on triplanar (axial, coronal, and sagittal) MRI scans, allowing for enhanced accuracy in volume quantification compared to traditional manual method. This approach significantly reduced the operator dependency typically associated with MRI analysis, facilitating a more objective assessment of tongue atrophy in patients. The implementation of AI not only streamlined the segmentation process but also demonstrated the potential for improved diagnostic capabilities in clinical settings.

**Fig. 1:**
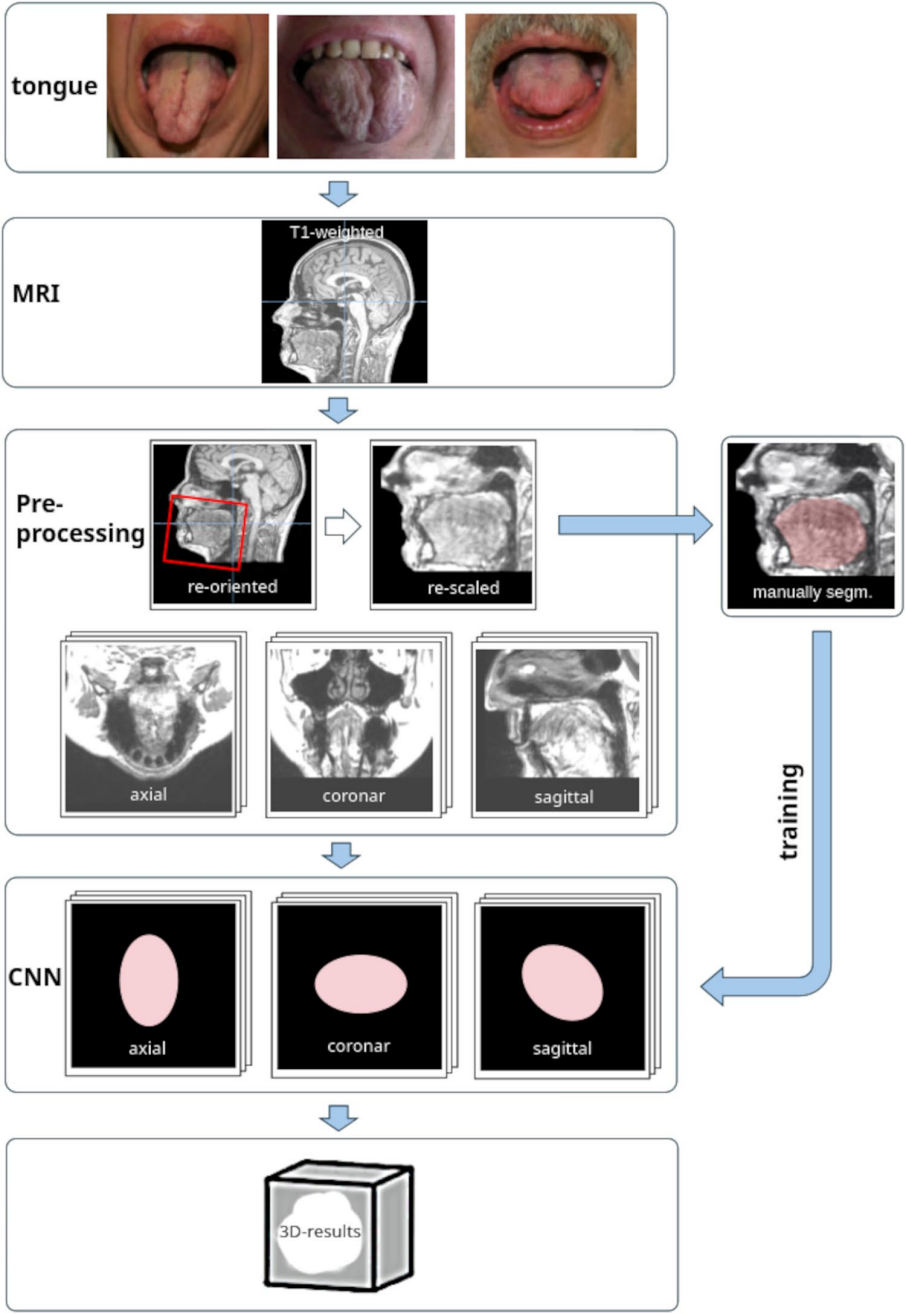
Schematic analysis cascade for tongue volume analysis. After preprocessing (re-orientation and re-scaling) of T1-weighted whole head recordings, stacks of axial, coronar, and sagittal slices were analyzed by a Convolutional Neural Network (CNN) [5]. The resulting predictions from each plane were then merged

The schematic analysis cascade for tongue volume analysis is shown in Fig. 1. In short: after preprocessing of T1-weighted whole head recordings, stacks of axial, coronar, and sagittal slices were analyzed by an operator-independent, triplanar CNN based on the U-Net architecture [5]. The resulting predictions from each plane were then merged using softmax averaging strategy. This method was developed and validated in a prior study which utilized identical MRI protocols in ALS [5]. For the specific quantification of tongue volumes in SBMA, the approach was applied to MRI data from 39 male patients diagnosed with SBMA and 51 age-matched male healthy control subjects.

Statistical analyses and data visualizations were performed in Python (version 3.11). Age-corrected tongue volumes were compared between SBMA patients and healthy controls using the Mann–Whitney U test. Correlations between age-corrected tongue volume and clinical parameters were assessed using Pearson’s correlation coefficient (r). For visualization, simple linear regression lines with 95% confidence intervals were fitted; the coefficient of determination (R^2^) was calculated. Statistical significance was set at p < 0.05.

## Results

In the 39 male patients with genetically confirmed SBMA who were included in the study, CAG repeat length ranged from 39 to 58, with a mean of 46 ± 4 repeats. Mean age was 58.3 ± 11.4 years. Of the 39 patients, two reported no bulbar symptoms with 20/20 in the bulbar SBMA-FRS subscore. Disease duration at study entry (reported as duration after first paresis) was 13 ± 8 years. Mean age at disease onset was 45.4 ± 11.5 years.

**Fig. 2:**
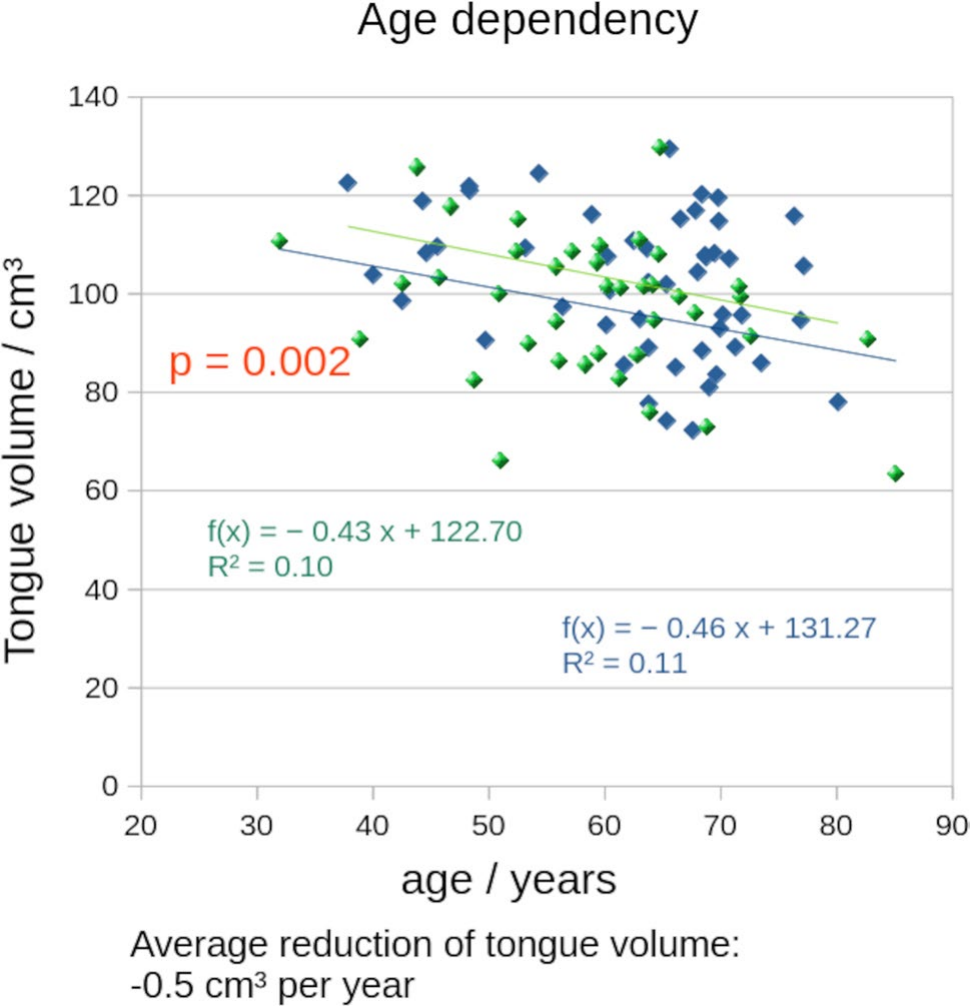
Age dependence of tongue volume for SBMA and controls. A significant age dependence (−0.5 cm^3^ reduction per year) was observed (p = 0.002). For the group comparison, tongue volumes were corrected for age. The straight lines represent independent two-parameter fits (slope, intercept) for the control group (blue) and the SBMA group (green). It should be noted that SBMA patients have a similar slope to the control group, but the y-intercept is different

The CNN-based automatic segmentation analysed tongue volumes in 39 participating SBMA subjects, compared to 51 healthy age-matched male controls (59 ± 19 years). Tongue volumes of the controls showed a significant decrease of the tongue volume with increasing age (r = −0.46, p = 0.002). Thus, tongue volumes of all subjects were corrected for age (Fig. 2).

**Fig. 3:**
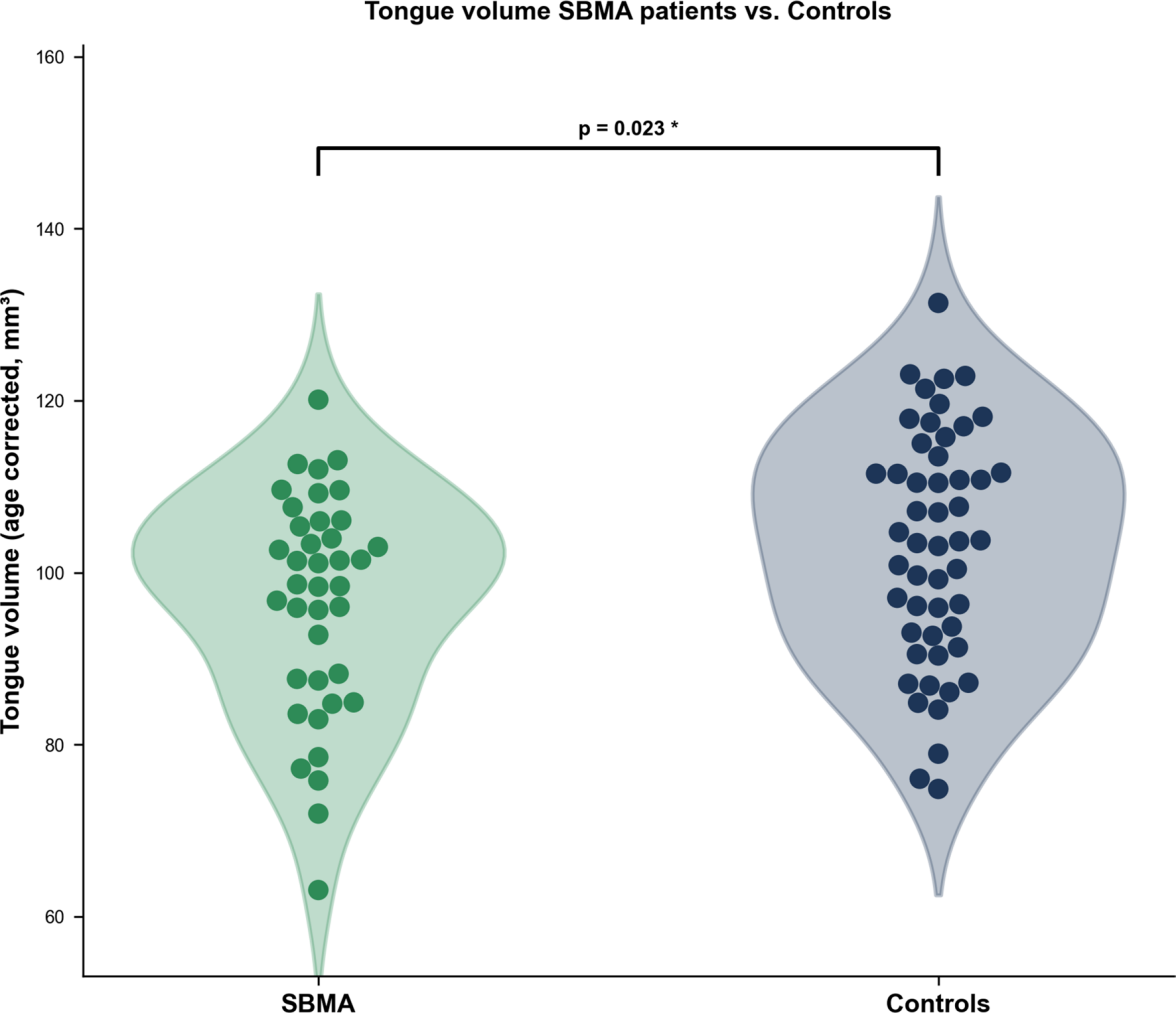
Tongue volume comparison between SBMA patients and healthy controls. Violin plot of age-corrected tongue volume (mm^3^) in SBMA patients (n = 39) and healthy controls (n = 51), with individual data points overlaid. Green: SBMA; blue: controls. p = 0.023 (Mann–Whitney U test)

**Fig. 4:**
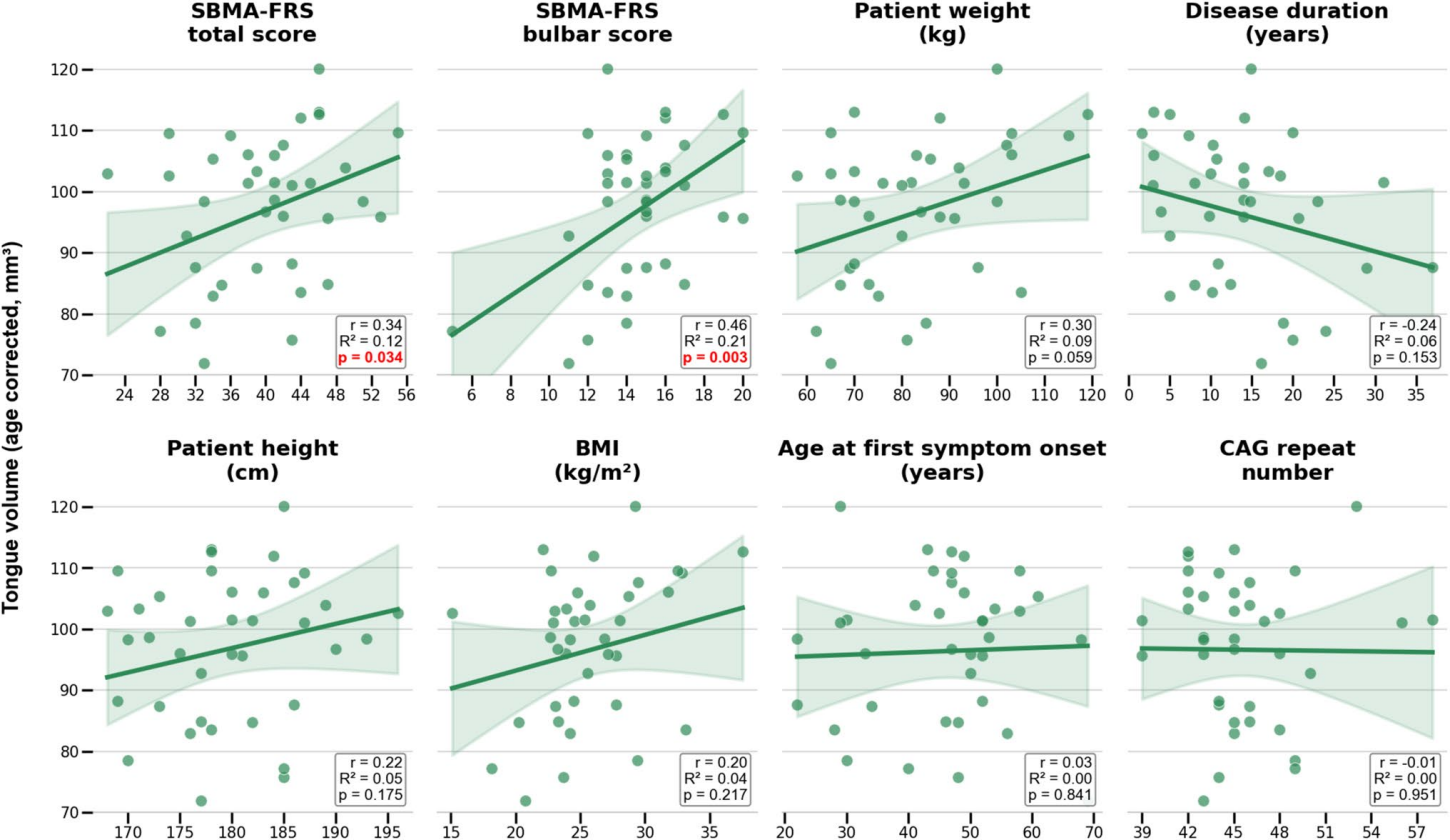
Correlation of age-corrected tongue volume with clinical parameters in SBMA patients. Each subplot shows individual data points, a fitted linear regression line, and the 95% confidence interval (Pearson correlation; n = 39). Abbreviations: SBMA-FRS, SBMA Functional Rating Scale; BMI, body mass index; CAG, cytosine-adenine-guanine

When comparing the the age-corrected volumes of the SBMA patient group to the control group, a significant atrophy of the tongue was observed in the SBMA cohort at the group level (p = 0.023) (Fig. 3). For clinical relevance, the tongue volumes were assessed across bulbar subscores of SBMA-FRS as well as total scores. Significant associations were observed for total tongue volume and total SBMA-FRS as well as bulbar SBMA-FRS subscore (p = 0.034 and p = 0.003, respectively). Correlations to other clinical or genetic markers like CAG repeat lengths, duration since first paresis, BMI, height and weight as well as age at first paresis were not significant (Fig. 4).

## Discussion

AI-assisted techniques utilizing CNN have emerged as a pivotal advancement in the accuracy of automatic tongue volume evaluation. In the current study, it could be demonstrated by their use that SBMA-associated atrophy of the tongue was significant at the group level when compared to controls and that atrophy correlated with total SBMA-FRS and with bulbar subscores. These results are in line with a previous study that showed significant reductions in tongue volume in patients with progressive bulbar palsy (PBP) compared to healthy controls (while no significant reduction had been observed in classical spinal ALS patients) [5]. In general, muscle imaging in MNDs, including SBMA, is notoriously underutilised and under-researched despite its pragmatic clinical value, biomarker potential and academic relevance [9]. It could, thus, be demonstrated in the current study that AI-based volumetry of the tongue, as a specific muscle with high clinical relevance, could identify patterns of atrophy linked to different MND variants, potentially enabling more tailored approaches to monitoring tongue volume/disease progression in future longitudinal studies, thereby enhancing the understanding and management of MND symptoms. The current investigation in SBMA adds to the study by Shaw and colleagues in individuals with ALS who used multi-contrast MRI for measurement of the volumes of key tongue muscles [6]. A study by Mano and colleagues demonstrated that tongue pressure was significantly correlated with the severity of dysphagia and dysarthria in SBMA patients [10], which could be a useful supplemental technique to make an individual prediction for risk of aspiration. Similar results were achieved when 39 SBMA patients underwent a videofluoroscopic swallowing study followed by maximal tongue pressure evaluation which was negatively associated with disease duration [11], but tongue muscle volumetry was not performed.

The relationship between tongue mechanics and swallowing efficacy further opens potential pathways for intervention in patients with MND. Parameters such as tongue pressure and endurance have been correlated with swallowing performance, emphasizing the need for targeted therapies, in line with data that tongue thickness in elderly is associated with maximal tongue pressure [12]. However, some studies suggest that tongue measurements alone may not be reliable predictors of swallowing impairments, indicating the complexity of correlations within clinical presentations [13]. Moreover, studies have explored the relationship between articulatory patterns and disease severity, demonstrating that changes in tongue function can serve as indicators of disease progression in MND [14, 15]. Additionally, the integration of neurophysiological techniques and morphological assessments has provided deeper insights into the functional impairments associated with MND [16, 17]. These studies about the functional impact of tongue alterations in MND underscore the need for future studies which correlate tongue function assessments (movements, swallowing, speech) with tongue morphology measurements. The lack of such specific functional assessments (beyond bulbar scores) is the major limitation of the current study.

In summary, the study employed advanced imaging methodologies to investigate the atrophy of tongue musculature in individuals affected by SBMA in correlation to clinical bulbar function, making this approach a potential biomarker for disease monitoring. SBMA is a unique MND, commonly associated with extra-neurological symptoms, such as cardiac and complex endocrine manifestations – these need careful screening and prompt management, making the timely recognition and thorough monitoring of SBMA particularly important [18]. Looking ahead, future studies should aim to establish clearer associations between tongue morphology and clinical outcomes in SBMA (and other neurodegenerative diseases). Investigating the relationship between specific tongue muscle changes and functional impairments will be crucial in elucidating the mechanisms underlying dysphagia and dysarthria in MND. Additionally, longitudinal studies are needed to explore the efficacy of targeted interventions based on structural and functional assessments of the tongue. Ultimately, a deeper understanding of these relationships might lead to improved management strategies for patients with respect to bulbar dysfunction.

## Data Availability

Reasonable data sharing requests can be addressed to the corresponding author and require a formal data sharing agreement with the University Hospital Ulm.
